# LigExtract: Large-scale Automated Identification of Ligands from Protein Structures in the Protein Data Bank

**DOI:** 10.1093/gpbjnl/qzaf018

**Published:** 2025-02-28

**Authors:** Natália Aniceto, Nuno Martinho, Ismael Rufino, Rita C Guedes

**Affiliations:** Department of Pharmaceutical Sciences and Medicines, Faculdade de Farmácia, Universidade de Lisboa, 1649-003 Lisboa, Portugal; Research Institute for Medicines (iMed.ULisboa), Faculdade de Farmácia, Universidade de Lisboa, 1649-003 Lisboa, Portugal; Department of Pharmacy, Phamacology and Health Technologies, Faculdade de Farmácia, Universidade de Lisboa, 1649-003 Lisboa, Portugal; Research Institute for Medicines (iMed.ULisboa), Faculdade de Farmácia, Universidade de Lisboa, 1649-003 Lisboa, Portugal; iBB─Institute for Bioengineering and Biosciences, Instituto Superior Técnico, Universidade de Lisboa, 1049-001 Lisboa, Portugal; Associate Laboratory i4HB─Institute for Health and Bioeconomy at Instituto Superior Técnico, Universidade de Lisboa, 1049-001 Lisboa, Portugal; Department of Pharmaceutical Sciences and Medicines, Faculdade de Farmácia, Universidade de Lisboa, 1649-003 Lisboa, Portugal; Research Institute for Medicines (iMed.ULisboa), Faculdade de Farmácia, Universidade de Lisboa, 1649-003 Lisboa, Portugal; Department of Pharmaceutical Sciences and Medicines, Faculdade de Farmácia, Universidade de Lisboa, 1649-003 Lisboa, Portugal; Research Institute for Medicines (iMed.ULisboa), Faculdade de Farmácia, Universidade de Lisboa, 1649-003 Lisboa, Portugal

**Keywords:** Ligand identification, Protein Data Bank, mmCIF, Data curation, Ligand clustering

## Abstract

The Protein Data Bank (PDB) is an ever-growing database of three-dimensional macromolecular structures that has become a crucial resource for the drug discovery process. Exploring complexed proteins and accessing their associated ligands are essential for researchers to understand biological processes and design new compounds of pharmaceutical interest. However, currently available tools for large-scale ligand identification fail to address many of the more complex ways in which ligands are stored and represented in PDB structures. Therefore, a new tool called LigExtract was specifically developed for the large-scale processing of PDB structures and the identification of their ligands. This is a fully open-source tool available to the scientific community, designed to provide end-to-end processing. Users simply provide a list of UniProt IDs, and LigExtract returns a list of ligands, their individual PDB files, a PDB file of the protein chains interacting with the ligand, and a series of log files. These logs record the decisions made during the ligand extraction process and flag additional scenarios that might have to be considered during any follow-up use of the processed files (*e.g.*, ligands covalently bound to the protein). LigExtract is freely available on GitHub (https://github.com/comp-medchem/LigExtract).

## Graphical abstract

**Figure:**
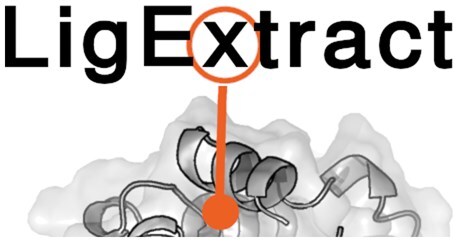

## Introduction

There is a large and ever-growing body of data in the Protein Data Bank (PDB; https://www.rcsb.org/) [1], and the ability to fully explore these data is crucial to draw insights regarding ligand binding to all sorts of biological targets. The sheer number of entries in the PDB (> 230,000 total structures and > 72,000 human structures as of January 2025) often requires automated processing and analysis of the deposited three-dimensional (3D) structural data, which is possible due to the highly structured and rich information within a PDB/mmCIF file. Previous examples of such large-scale analyses enabled by automated PDB mining include studies on binding site comparison [2,3], promiscuity and druggability investigations [4], and binding mode prediction [5]. The ability to automate the mining of data in the PDB has also generated other important databases and tools, such as KLIFS [6], PocketDB [7], ProteinsPlus [8], sc-PDB [9], or Binding MOAD [10].

Whether the goal is to study ligands or binding sites, or to retrieve a list of holo and apo structures for a given target, these tasks rely on the ability to locate ligands with pharmaceutical interest within PDB structures. Even though all molecules can be of theoretical pharmaceutical interest (including ligands such as ions or lipids), most drug development studies focus on organic molecules that bind a target to elicit an effect of pharmaceutical/medical nature. For this reason, we focus on identifying ligands of this nature, and refer to them as “ligands of interest”. To identify such ligands, most studies operate under the assumption that they belong to the HETATM group in the *_atom_site* category of the mmCIF file, which is where ligands are typically expected to be found. Ligands are also generally associated with a unique ligand code (historically a 3-character code, and now a 5-character code). This is true for a large number of PDB structures. However, due to the diverse nature of biological complexes deposited in the PDB, there are multiple instances of ligands that are not annotated in this typical format. For example, peptide-like ligands may appear entirely in the ATOM group or partly in the ATOM group and partly in the HETATM group (*e.g.*, 5LF6) within *_atom_site*. According to data from the Biologically Interesting Molecule Reference Dictionary (BIRD; https://www.wwpdb.org/data/bird), there are nearly two thousand different peptide-like and oligosaccharide compounds in the PDB. While this library of atypical ligands is helpful in facilitating the unambiguous identification of large-molecule ligands, it is currently not exhaustive, as it misses the peptide ligand of 5LF6, for example.

**Figure 1:**
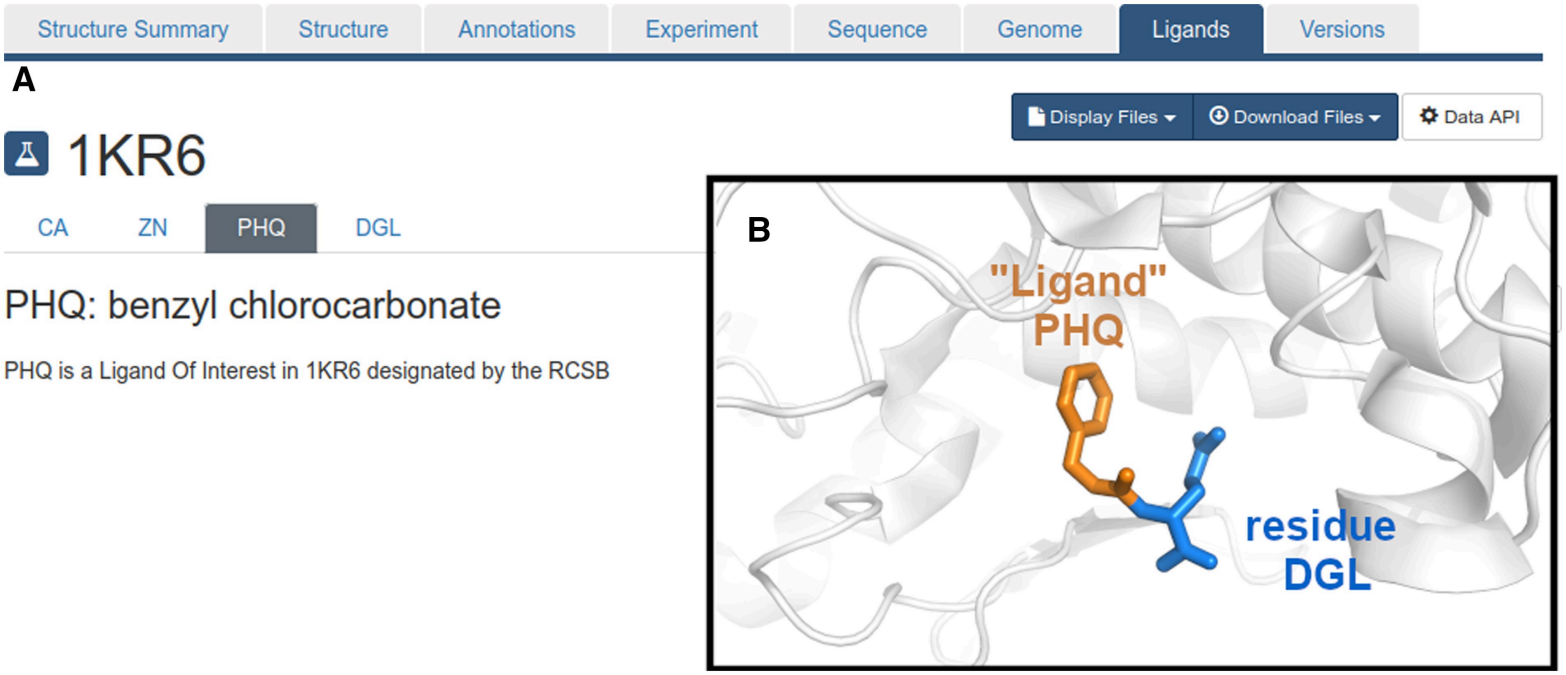
An example of an apparent ligand (PHQ) that is actually a part of a larger molecule **A**. PHQ is annotated as a ligand of interest in PDB 1KR6. This image is a snapshot from https://www.rcsb.org/ligand-validation/1kr6/PHQ, captured in January 2025. **B**. PHQ (orange) is a fragment of the true ligand (benzyloxycarbonyl-D-glutamic acid) in this PDB entry and is connected to an additional amino acid residue DGL (blue).

The inability to locate various atypical ligands with a more challenging type of annotation may have important consequences, such as the misclassification of a protein as being in the apo state, the accidental exclusion of certain ligands from an analysis or dataset, or the inclusion of ligands with incomplete structural data. Furthermore, to add to the complexity of identifying ligands within a PDB structure, solvent molecules or crystallization additives are annotated in the same way as molecules with pharmaceutical interest, receiving a unique ligand code and being found in the HETATM group. Ultimately, this contributes to skewing analyses and limiting the accuracy or completeness of their resulting conclusions. Recently, the PDB platform implemented an additional annotation of “Ligand of Interest” (LOI), enabling a more straightforward identification of ligands of interest among a very large collection of ligand-like molecules. The PDB 1O8V is an example where this annotation is present. While this is an important step toward a clear and simple identification of ligands of interest, it is not yet found in many PDB entries, and thus some of the more complex issues mentioned above remain unresolved. For example, PHQ (benzyl chlorocarbonate) appears in PDB 5LF6 as part of a larger peptide ligand (Z-LLY Ketoaldehyde [11]), while in PDB 1KR6 it appears as a standalone ligand of interest (as shown in https://www.rcsb.org/ligand-validation/1KR6/PHQ), despite being merely a fragment of the full-length inhibitor molecule (benzyloxycarbonyl-D-glutamic acid), as shown in **Figure 1**.

The LOI annotation is intended to be provided by depositors, as implied by the description of *_pdbx_entry_details.has_ligand_of_interest* on https://mmcif.wwpdb.org: “*A flag to indicate if author has indicated that there are any or no ligands that are the focus of research.*” This annotation is optional, and as of January 2025, only 22.1% of PDB entries have this annotation, according to https://mmcif.wwpdb.org/dictionaries/mmcif_pdbx_v50.dic/Items/_pdbx_entry_details.has_ligand_of_interest.html.

Multiple tools are available for automated, semi-automated, or manual large-scale ligand identification, as listed in **Table 1**. However, these tools exhibit different limitations, which have been addressed in the design of LigExtract and are explained in the following sections.

**Table 1:** Summary of existing ligand extraction tools

Tool	Command line	Refs.
PDBconv (IChem)	Yes	[12]⁠
Binding MOAD	No	[10,13]⁠⁠⁠
MOAD Ligand Finder	Yes	https://github.com/lucagl/MOAD_ligandFinder
CCD	Yes	[14]⁠
Protoss (ProteinsPlus)	Yes	[15,16]⁠
PLIP	Yes	[17,18] ⁠
BioLiP2	Yes	[19,20]⁠ ⁠

PDBconv relies on a pre-assembled list of ligand codes to identify ligands [12], which is a limited approach given that some ligands are not encoded under a simple ligand code, as mentioned earlier. The Binding MOAD (https://bindingmoad.org/) [13] and its associated GitHub tool MOAD Ligand Finder (https://github.com/lucagl/MOAD_ligandFinder) appear to be one of the only tools capable of locating peptide ligands that are found exclusively in the ATOM group (*e.g*., chains E and F in 1OKV). However, they exclude covalent ligands, peptide ligands above a certain size, and PDB structures above 2.5 Å resolution, among other criteria. Moreover, MOAD relies on manual annotation, which further limits its usability, and it also excludes other types of less typical ligands, as we will discuss later. Notably, during the peer-review process, MOAD was discontinued, which presumably means no future data will be curated and added into it. The Chemical Component Dictionary (CCD; http://www.wwpdb.org/data/ccd) [14] is a reference file with all residue codes currently in the PDB (here, “residue” is used as per the terminology of PDB and simply refers to a set of atoms, which may be in a protein, of peptide-like nature, or not a peptide at all). CCD identifies ligands by their unique ligand codes and therefore shares the same shortcoming as PDBconv. Ligand Expo (formerly Ligand Depot; http://ligand-expo.rcsb.org/ld-download.html) [21], one of the resources available to access CCD information, provides downloadable files with the correspondence between PDB entries, ligand codes, and ligand counts. However, this will soon be decommissioned, and the LigExtract code will be adjusted accordingly.

Despite not being built with the main purpose of ligand identification, ProteinsPlus (https://proteins.plus) [8,22] has a REST service that allows automating the use of Protoss (https://proteins.plus/help/protoss_rest) [15,16] to identify ligands. ProteinsPlus is currently one of the most sophisticated tools for this use, as it appears to employ more complex heuristics to address issues such as ligands split between the ATOM and HETATM groups. However, this tool is still unable to identify ligands in different scenarios of added complexity. At the same time, even when this tool is able to detect some of the more challenging ligands, it frequently also identifies molecules that aren’t organic compounds of pharmaceutical interest.

PLIP (https://plip-tool.biotec.tu-dresden.de/plip-web/plip/index) [17,18] is also not primarily designed for ligand identification but can be used as the first step to automatically identify ligands. However, this tool simply identifies any atypical residue as a ligand (*i.e.*, ranging from mutated protein residues to ligand and solvent residues) and considers each individual residue as a separate ligand (here, “residue” refers to a group of atoms and not necessarily an amino acid residue, in line with the PDB terminology). Thus, PLIP could be used, at most, to shortlist potential small-molecule ligands, leaving out (partly or entirely) ligands composed of amino acids.

Lastly, BioLiP2 [19,20] (also referred to interchangeably as BioLiP) is a semi-automatically assembled database and, to our knowledge, the resource most similar to LigExtract. This, alongside MOAD, appears to be the only tool capable of locating peptide ligands that are found exclusively in the ATOM group. They classify a ligand as being of biological interest if it belongs to the HETATM group and does not belong to their “artifact list”. Ligands found among the artifacts are reclassified as a ligand of interest only if they are mentioned (directly or under a synonym) in the original publication’s abstract. One of the main differences between BioLiP2 and LigExtract is that BioLiP2 only considers peptide ligands up to 30 residues. Even though synthetic peptides are commonly under this size, multiple false positives may arise from complexes containing naturally small proteins, such as thrombin light chain (1OYT), humanin (7WVX), and Protein transport protein Sec61 subunit beta (8B6L). In fact, as of 19 February 2024, UniProt contains nearly 6000 reviewed proteins with up to 30 residues in length, of which 160 already have 3D structures. All these proteins could potentially be misclassified as peptides by BioLiP2. The opposite scenario — false negatives — can also occur due to the size cutoff, as larger peptide ligands (*e.g.*, semaglutide in 7KI0 or teriparatide in 7VVN) will be missed. In contrast, LigExtract employs a more conservative and nuanced approach, which will be detailed in the Results and discussion section.

Shao et al. [23] recently published work on analyzing the quality of ligands where they also highlighted some issues related to the ability to identify ligands; however, they focused mostly on small-molecule ligands. This is another example of a study where authors would have benefited from a tool that identifies ligands at a large scale.

Facing the current paradigm where large-scale ligand identification in PDB data is hindered by multiple challenges, we were prompted to create LigExtract, a tool that allows locating and extracting ligands of different types in virtually any PDB/mmCIF structure. It is important to note that, because we have not tested this tool beyond human structures, we prefer to describe its applicability more conservatively (*i.e.*, applied to human structures). However, there is no obvious reason why LigExtract could not be applied to non-human structures. In theory, LigExtract can be applied to all structures in the PDB.

LigExtract provides an alternative solution to cope with the complexity of ligand annotation in PDB structures, addressing both typical non-covalent small-molecule ligands and more atypical ligands such as covalent modulators, peptides, peptide-like molecules, and oligosaccharides. In this study, we provide an overview of the LigExtract tool, accompanied by benchmark studies to demonstrate its functionality. LigExtract can be installed and run from the command line via its GitHub repository (https://github.com/comp-medchem/LigExtract). Currently, LigExtract is exclusively available as a command-line tool.

## Method

### Benchmark datasets of PDB structures and manually assigned ligands

A “challenging*”* dataset of 107 PDB structures covering a variety of scenarios was assembled manually to benchmark ligand extraction performance (available in Table S1, where the included or excluded ligands are annotated in detail for each PDB). These PDB structures correspond to 18 protein queries and each PDB is associated with a single protein query. This dataset was mostly populated with PDB structures that represent challenging ligand extraction cases, which we encountered during the development of the LigExtract tool. However, some straightforward extraction cases were also included (*i.e.*, proteins with only one ligand found in the HETATM group or proteins with no ligands).

This dataset was manually annotated with all ligands of interest after visually inspecting each entry on the PDB platform and its corresponding publication (Table S1). As each PDB corresponds to a single protein query, we only considered ligands that are in contact with the query protein in each case. This is another source of complexity that remains unaddressed when the goal is to assemble ligands for a given protein of interest. A small subset of this dataset was also used for in-depth manual inspection of the selected ligands and their structures by LigExtract *versus* MOAD and ProteinsPlus. The full list of PDB IDs that constitute this dataset is: 1A2C, 1A3B, 1A4W, 1ABI, 1ABJ, 1AD8, 1AI8, 1B7X, 1BA8, 1BHX, 1BTH, 1C1U, 1C1V, 1C1W, 1C4U, 1C4V, 1CA8, 1D6W, 1DE7, 1DIT, 1DM4, 1DX5, 1E0O, 1EB1, 1EOJ, 1EOL, 1FPC, 1FPH, 1G30, 1G37, 1MOX, 1NM6, 1NRR, 1NRS, 1NU7, 1NU9, 1NY2, 1NZQ, 1O0D, 1OOK, 1OYT, 1PPB, 2GW2, 2P64, 2WFI, 2WFJ, 2Z62, 2Z65, 3CRG, 3CTT, 3DA9, 3ILR, 3IN9, 3INA, 3JUT, 3K1X, 4MI5, 4NEU, 5FV8, 5GSA, 5H14, 5H15, 5H17, 5H19, 5H24, 5HG5, 5HG7, 5HG8, 5HG9, 5HGG, 5HGV, 5HX6, 5HYN, 5LE5, 5LEX, 5LEY, 5LEZ, 5LF0, 5LF1, 5LF3, 5LF4, 5LF6, 5LF7, 5T01, 5WG6, 6M90, 6M91, 6M92, 6M93, 6NW2, 6NYH, 6Y3V, 7KME, 8KME, 1GY3, 2R3J, 5ANK, 3DOG, 5LS6, 4Y8L, 4QLQ, 4R00, 1QMZ, 4KXA, 4KX8, 6FUV, and 4KX9.

An “easy*”* dataset of 25 PDB structures was also manually assembled (Table S2) to benchmark ligand extraction performance. This covered 24 protein queries where each PDB was derived from a single protein query. The full list of PDB IDs that constitute this dataset is: 4O91, 6AJZ, 1S9J, 1E8Z, 3ALN, 6AQB, 4GS6, 3SXR, 5HES, 2VO0, 2ANY, 1P57, 5FHT, 6TS6, 3NCL, 3GTA, 3N80, 1G8I, 1OIZ, 4MWI, 4BCX, 1G1Q, 3RIP, 3MS6, and 3S91.

Finally, a third “random” dataset (Table S3) was assembled by randomly selecting 20 proteins from a group of 673 proteins with well-established druggability (taken from T_clin_ and T_chem_ in Oprea et al. [24]). These proteins were processed by LigExtract at its current state of development within the context of a separate project. For each of the 20 selected proteins, a random PDB was chosen and manually annotated with ligands based on visual inspection of the corresponding PDB online entry and its publication. The full list of PDB IDs that constitute this dataset is: 3MWI, 3NBW, 3UT1, 4EHR, 6C1B, 4KMY, 2NN7, 2J4A, 3ZKI, 6UZT, 3PLS, 2F1Y, 6E69, 4AW1, 2I4Z, 3DWB, 3V8W, 2H5J, 5G1X, and 4J1E.

### Performing ligand extraction with ProteinsPlus

To benchmark the ligand extraction ability of ProteinsPlus, we processed the PDB entries from the three benchmark datasets through ProteinsPlus using the Protoss REST service following the instructions in https://proteins.plus/help/protoss_rest. For each PDB code query, the resulting SDF file in the “ligands” field was downloaded, and all ligand names were extracted from the SDF file. The ligand names were transformed to match the naming system of the benchmark datasets, enabling direct comparison between different ligand extraction tools. ProteinsPlus encodes ligands following the format “*residueCode_chain_residueNumber*”. If a ligand consists of more than one residue, the multiple residues are concatenated with “_” symbols (*e.g.*, “NAG_G_2_BMA_G_3_FUC_G_4_NAG_G_1”). Therefore, if a ligand name contains more than three “_” symbols, it is identified as a multi-residue ligand, here termed as “chain ligand” (*i.e.*, encoded as a chain in the PDB record rather than as a small-molecule entity). The full process was automated with Python.

All PDB entries in our manually assembled datasets were successfully queried in our automated retrieval process. This is important to check, as a PDB with no ligands would produce the same outcome as a failed query for that PDB (*i.e.*, no ligands in the output).

### Performing ligand extraction with MOAD

To benchmark the ligand extraction ability of MOAD, we queried the MOAD database with the PDB entries from the three benchmark datasets. To do so, the entire MOAD database was downloaded from https://bindingmoad.org/Home/download (note: this resource has been discontinued and is no longer available) in the form of the *every.csv* file. This file was downloaded on 22 April 2022 by accessing “*Binding data*” under “All of Binding MOAD”; however, the file has not changed up to the date when the experimental work stopped on 2 December 2024. The ligand names were converted to match the naming system used in the benchmark datasets to enable direct comparison between different ligand extraction tools. MOAD encodes ligands using the format “residueCode:chain:residueNumber”. If a ligand consists of more than one residue, the multiple residue codes are concatenated with spaces before the first “:” symbol, *e.g.*, “34H LEU PRJ OAR:J:1”. Thus, whenever more than four characters are present in the string before the first “:” symbol, the corresponding ligand is classified as a chain ligand. Additionally, only ligands with a “valid” annotation in the MOAD database file were retained.

There was an incomplete overlap between the PDB entries in our manually assembled datasets and those retrieved from MOAD, indicating that not all PDB entries in the three benchmark datasets exist in the MOAD database.

### Performing ligand extraction with BioLiP2

To benchmark the ligand extraction ability of BioLiP2’s workflow, we queried the BioLiP2 database with the UniProt IDs from the three benchmark datasets and filtered each query for the particular PDB IDs covered in our datasets. This was done by manually searching each UniProt ID on BioLiP2’s web platform (https://zhanggroup.org/BioLiP/index.cgi#by_name) and downloading the complete results in .txt file obtained from each query. After collating all results, we processed them to convert the ligand information into the format used in the benchmark datasets. If an identified ligand is not a small molecule (*i.e.*, is annotated as “peptide” or “dna” in our subset of queries), it is renamed as “chain-” followed by its chain ID assigned by BioLiP2 (*e.g.*, “chain-B”). Otherwise, small-molecule ligands were identified by their PDB ligand codes. We verified that all receptor chains in the final set of ligands corresponded to a UniProt–PDB pair in the benchmark datasets.

### Benchmarking ligand extraction performance

To benchmark ligand extraction performance, the ligands extracted from BioLiP2, MOAD, and ProteinsPlus (via Protoss) were compared with those extracted by LigExtract. Performance was assessed using the *cluster* mode (details of the algorithm are provided in the Results and discussion section), as this mode most closely resembles the comparative tools used in the benchmarking (*i.e.*, all ligands are retained without deduplication of possibly equivalent ligands). Performance was evaluated in terms of precision and recall as defined in Equations 1 and 2. Both metrics range between 0 and 1, where 1 indicates maximum performance.


(1) $$\mathrm{Precision}=\frac{\left|\mathrm{detected}\;\mathrm{ligands}\cap \mathrm{true}\;\mathrm{ligands}\right|}{\left|\mathrm{detecte}d\;\mathrm{ligands}\right|}$$

(2) $$Recall=\frac{\left|\mathrm{detected}\;\mathrm{ligands}\cap \mathrm{true}\;\mathrm{ligands}\right|}{\left|\mathrm{true}\;\mathrm{ligands}\right|}$$

As mentioned earlier, we acknowledge that all molecules binding to a protein can be defined as ligands; however, the goal of this tool is to identify organic compounds of pharmaceutical interest (especially because identifying other types of molecules, such as ions, is straightforward and already achievable). Therefore, to avoid any confusion with the term “ligand” (which in the context of the PDB refers to any type of small molecules), we will use the terms “true ligand” for molecules of pharmaceutical interest and “non-ligand” for other molecules, such as solvents, ions, and crystallization additives. Therefore, in Equations 1 and 2, *detected ligands = true ligands + non-ligands*, as detected by a given tool. If one is interested in finding ligands under a broader definition (*i.e.*, modulator or any kind), BioLiP2 currently applies a heuristic to identify such ligands.

When no true ligands exist, the resulting recall is considered invalid (*e.g.*, *numpy.nan*), since there are no ligands to retrieve and the calculation would involve division by zero. Conversely, when no ligands are detected (*i.e.*, zero detected ligands), it is not appropriate to report precision as 0, since this reflects a recall failure rather than misidentification; in such cases, precision is also considered invalid (*numpy.nan*). An exception to these two scenarios occurs when there are zero detected and zero true ligands, which is reported as precision = 1 and recall = 1, indicating a perfect match between actual and detected ligands. Finally, the average precision and average recall values (across all benchmark PDB entries) are calculated to yield a single global precision and a single global recall that can be easily compared between tools.

## Results and discussion

### Description of the LigExtract tool and its functionalities

**Figure 2:**
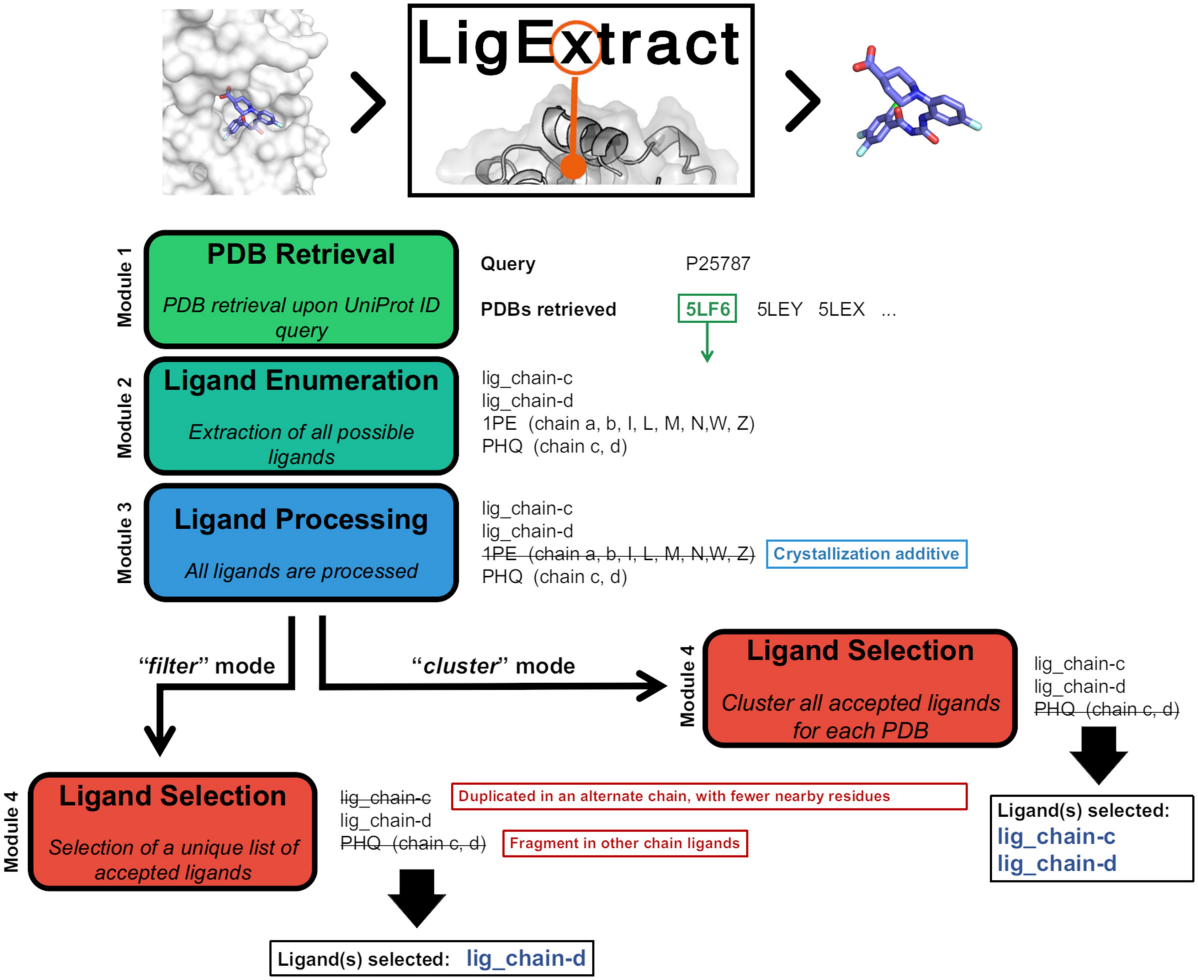
The LigExtract workflow applied to an example PDB (5LF6) In LigExtract’s workflow, Ligand Selection is bifurcated into two mutually exclusive modes: the *filter* mode and the *cluster* mode. In this example, PHQ may be mistaken for an individual ligand (as it is labeled as HETATM and not a mutated residue), but it is actually a part of a peptide-like ligand.

LigExtract is structured into four modules: PDB Retrieval, Ligand Enumeration, Ligand Processing, and Ligand Selection. Each module generates a log file that users can inspect to get detailed information regarding events encountered and decisions made throughout the workflow. The LigExtract workflow starts with a list of UniProt ID queries provided by users and finishes with a list of eligible ligands for each PDB corresponding to each UniProt ID. Additionally, users must define the maximum value of acceptable resolution to be considered by LigExtract. The list of UniProt IDs and the resolution limit are the only inputs that users need to provide. A summary scheme of the LigExtract workflow is shown in **Figure 2**. Currently, LigExtract does not consider structures without resolution data and considers the highest resolution when multiple values are reported within the same PDB record.

Prior to the main four-module workflow, a one-off preparation step is performed, which users need to run only once, regardless of how many times the workflow is used. This step should be repeated only if users wish to update some of the retrieved or processed files. Some of the main tasks performed during this step include: (1) downloading the complete list of currently available PDB entries; (2) downloading the complete list of PDB chain-to-UniProt ID mappings for all existing PDB structures; (3) processing the mmCIF file with all entries in the Biologically Interesting molecule Reference Dictionary (BIRD) [25], available for download at https://www.wwpdb.org, to generate a list of PRD ID-to-PDB mappings and a list of all PRD ID sequences (PRD ID is the identifier in BIRD); (4) downloading the ligand counts file from Ligand Expo; and (5) downloading BeEM [26]. As Ligand Expo will be discontinued, we will implement our own procedure to obtain the information in these files to eliminate dependency on Ligand Expo. For future reference, mmCIF files are organized into categories, and each category has various items.

Although LigExtract is theoretically applicable to all protein structures, it has been extensively tested on human structures and only briefly tested on non-human structures.

Following this preprocessing step, we will describe in detail the four modules in LigExtract.

#### Module 1: PDB Retrieval

This module automates the retrieval of all PDB entries associated with the user-supplied list of UniProt IDs. This was originally achieved by the automated mapping of UniProt IDs to PDB entries using the REST API mapping tool available at https://www.uniprot.org (Python v3.0) that performs UniProt-to-PDB mapping. However, the mapping provided by UniProt fails to retrieve certain PDB entries (including older entries with 5-character ligand codes). For example, in the case of Q9NUW8, UniProt maps 42 PDB IDs, but a direct search within the PDB retrieves 48 structures. Alternatively, LigExtract employs the chain-to-UniProt ID mapping provided by Structure Integration with Function, Taxonomy and Sequence (SIFTS; https://www.ebi.ac.uk/pdbe/docs/sifts/index.html), which is a project from the Protein Data Bank in Europe (PDBe) team and offers a chain-to-UniProt remedial mapping (achieved through sequence matching) publicly available for download.

The mapping process considers the maximum resolution value set by users. Following the completion of UniProt-to-PDB mapping, all mmCIF files (in *.cif format) corresponding to all retrieved PDB entries are automatically downloaded in batch using the PDB’s script available at https://www.rcsb.org/docs/programmatic-access/batch-downloads-with-shell-script. To prevent redundant downloads upon rerunning a job, already downloaded PDB entries are excluded from the list of retrieved PDB entries prior to batch downloading. All mmCIF files were converted to *.pdb format using BeEM [26]. To address the implementation of 5-character ligand codes, we convert these into the corresponding short codes provided by BeEM and apply this conversion to the mmCIF files.

To properly consider PDB files annotated with what are now secondary UniProt IDs (*i.e.*, outdated UniProt IDs), all UniProt IDs in all retrieved PDB files are mapped to their current primary UniProt IDs. Cases where PDB files contain secondary UniProt IDs happen when PDB entries were deposited prior to changes in UniProt IDs (*e.g.*, the merging or de-merging of old entries) recorded within the PDB files. This mapping is crucial to correctly identify the chains that are of interest to users, avoiding accidental exclusion of chains of interest and consequently of their accompanying ligands.

An important consideration is how LigExtract determines whether a PDB file contains meaningful records for the protein of interest. The PDB deposition guidelines state that “*Each chain for which there is an appropriate sequence database reference will have DBREF, _struct_ref, and _struct_ref_seq records. When no sequence database reference is available the sequence will be self-referenced (i.e.*,* the database reference will be the PDB entry itself).*” Therefore, LigExtract assumes that a self-referenced chain indicates one of two scenarios: (1) it is a protein that could not be mapped to any UniProt ID at the time of deposition, or (2) it is a legitimate peptide (not a protein fragment captured in the crystal). Therefore, remediation applied to these chains is non-trivial, as demonstrated in the case of 5HGV. In this structure, a protein (OGT) is complexed with a small peptide (chains B and D). This peptide corresponds to a 13-residue portion of the CSNK2A1 protein, but it was intentionally synthesized at this length (*i.e.*, it is not a portion of a longer protein chain). If the goal is ligand extraction, mapping chains B and D in 5HGV to the CSNK2A1 protein would be misleading, as these are peptides used as ligands in the experimental setting. However, the chain-to-UniProt mapping by SIFTS maps those two chains in 5HGV to a UniProt ID.

Considering that both using and ignoring SIFTS mapping could lead to mislabeling of the chains across PDB entries, LigExtract employs SIFTS mappings to allow capturing as many protein instances as possible in response to a UniProt query, as many legitimate proteins lack UniProt annotation in their deposited PDB files (*e.g.*, 6FUV, in which the sole protein chain has no UniProt ID). To mitigate any undesired labeling of peptide ligands, some steps are taken later in the workflow to reclassify protein chains that are peptide ligands. Nevertheless, as will be discussed in the benchmarking studies, such identification is complex and would likely require some form of natural language processing applied to the original publication (and even beyond that).

The entire ligand extraction process is logged in a global log file, which users can inspect and parse to validate the automated decisions made by LigExtract. Additionally, in this module, individual log files for each PDB are also produced. These log files contain information about covalent bonds that may exist between ligands and proteins, including the precise atoms involved in these bonds. This is accomplished by inspecting the *_struct_conn* section of the mmCIF file and identifying any instances where protein–nonprotein pairs are present as “covale” in the *conn_type_id* field.

The log files also flag ligands that are present in a large number of PDB entries, which could be indicative that a particular ligand is not a ligand of interest but rather a common crystallization additive.

#### Module 2: Ligand Enumeration

This module iterates through each PDB entry and executes a procedure to identify all possible ligands that may exist within it. As LigExtract is developed to employ as few assumptions as possible, it considers small molecules, peptides, and large molecules (such as antibodies). All assumptions that are applied are described throughout this document. Keeping assumptions to a minimum is important, as researchers might seek different types of binding information, and it is much easier to have different types of ligands and afterward, employ a simple size filter to obtain a particular type of ligands.

Peptide-like ligands are assembled by considering chains not associated with any UniProt ID. If a chain is mapped to a UniProt ID, a set of rules is applied to attempt to reclassify it as a peptide ligand derived from a protein. To allow for a more conservative approach, *_entity.type* = “polymer” and *_entity.src_method* = ”syn” are necessary conditions but not sufficient. Chains that fall under this annotation will be considered as ligands if (1) their actually resolved sequence in the PDB is not considerably shorter than the theoretical sequence annotated by the authors, and (2) the theoretical sequence annotated is much shorter than the full-length (reference) sequence in UniProt. This rationale is based on the expectation that a peptide derived from a protein will intentionally have a much shorter sequence in the PDB than that in UniProt, and that its residues will be almost completely present in the modeled structure.

To ensure complete assembly of the ligand, bonds in the *_struct_conn* section will be searched to find residues belonging to the HETATM group that are connected to residues in the reference chain. To aid this process, LigExtract compares all possible ligand chains with sequences in the BIRD ligands collected during the preprocessing step described above, and full matches are used to confirm a chain as a ligand.

Ligands present in more than 250 PDB entries are flagged as potentially not being a ligand of interest in the log file (*i.e.*, the “frequent ligand rule”), unless they are found in a list of ligands to be retained (which includes ligands such as ATP, which are very common but nevertheless legitimate ligands). This list was manually assembled (*n* = 15) by us and is available in the GitHub repository (https://github.com/comp-medchem/LigExtract/tree/main/benchmark_data). It is composed of examples we encountered from manual inspection of hundreds of PDB entries during the design of LigExtract and, therefore, is not exhaustive. Nevertheless, this list is not expected to be much lengthier than its current state since ligands of pharmaceutical interest that are simultaneously present in hundreds of PDB entries are not a common occurrence. The use of such a list prevents excluding very common ligands when the “frequent ligand rule” is applied. We currently have no update plan in place for updating this list, as the ligands it contains are not common and are not expected to increase dramatically overtime, but we will update the list as we encounter more cases of these “frequent ligands”.

Additionally, ligands with less than 3 heavy atoms or with more than 10 molecules are excluded in this module, considering that these conditions are not expected to be associated with ligands of interest.

This module also handles chain ligands that are oligosaccharides, by analyzing the *_pdbx_entity_branch*, *_struct_conn*, and *_pdbx_branch_scheme* categories to rule out any possible glycosylation groups. One case where an oligosaccharide is an important ligand is 4RDA, in which amyloid-like protein 1 is complexed with a heparin dodecasaccharide (chains C and D). Finally, chains that are annotated with the term “non-polymer” or “macrolide” in *_entity.type*, will be considered ligands.

During this step, ligands are saved in their respective individual files. Note that covalent ligands are stored with their covalent structures. If necessary, users need to revert these ligands to their original non-covalent form after LigExtract has finished processing. Users can know exactly which ligands need to be repaired, from the log files.

#### Module 3: Ligand Processing

This module removes all ligands with a code present in a manually assembled exclusion list of ligands (available in the GitHub repository), which is composed of solvents, crystallization additives, and salts. This list was compiled from a variety of resources that list common reagents used in crystallography [27–31], as well as from manual inspection of hundreds of PDBs and their associated publications during the development of LigExtract. This is a conservative list, and additional ligands are removed through different and more specific filters described in the following sections. In this step, all PDB entries that do not have the queried UniProt ID (or its secondary UniProt IDs) in any chain in the *_struct_ref_seq* category are removed. This step is crucial, as certain deposited structures may be associated with a small portion of the desired protein and play no role in ligand binding within that PDB record.

In the final step of this module, all residues within 6 Å of each ligand are collected, and ligands that are not close to at least one residue belonging to the chain of the UniProt ID query are discarded. Each ligand that survives this filter is listed in a pocket information file, annotated with its filename, a list of all nearby residues, their respective chain, and the residue count.

#### Module 4: Ligand Selection

This module can be operated under two modes: *filter* and *cluster*. The *filter* mode is a more assertive mode that identifies the most probable ligand and returns its PDB file, with the 3D coordinates extracted from the original PDB file, as is, accompanied by a series of log files listing the additional molecules that are excluded during the selection process and their exclusion criteria. The *cluster* mode is a more cautious mode that returns all ligands in separate files, accompanied by a log file that provides the frequency of each ligand across PDB entries, which assists users in manually selecting the correct ligand.

This module checks whether any of the small-molecule ligands are actually part of a larger ligand. In such cases, the smaller ligand is removed, and this action is recorded in a log file.

Using the more assertive *filter* mode, all ligands that reach this stage undergo a deduplication step that selects only one ligand per unique ligand structure. For example, multiple ligands with the same ligand ID but located in different protein chains (*e.g.*, 5U6.A, 5U6.B, and 5U6.C) are compared, and the ligand with the highest number of nearby residues is selected. For multi-chain ligands, one way to easily determine if they are equivalent is by inspecting the chains annotated under the same *_entity.id*. Similarly to small-molecule ligands, when two or more chain ligands correspond to the same structure, the ligand with the highest number of nearby residues is selected.

LigExtract does not assume that a structure deposited under a given PDB ID must have only one ligand and, indeed, examples of such a scenario are shown in **Table 2**. Instead, LigExtract reports all ligands that pass all filters and checks as equally likely ligands. Additionally, many structures have bound ATP or ADP molecules, which, depending on the specific research context, might also be considered ligands. We believe that it is more useful to let users employ an *ad-hoc* filter to exclude ligands such as ATP or antibodies, rather than assuming that no user will want to consider these molecules as a ligand. This approach ultimately makes LigExtract more versatile.

**Table 2:** Examples of challenging PDB structures

UniProt query	PDB ID	Ligand comment	Challenge	LigExtract	ProteinsPlus	MOAD	BioLiP2
P00800	1KR6	An apparent ligand (PHQ) is actually a group in a larger ligand structure (PHQ + DGL, namely benzyloxycarbonyl-D-glutamic acid)	Exclude PHQ and reassemble the 2-residue ligand	**Small “apparent ligand” removed**; **2-residue ligand reassembled**	**Small “apparent ligand” removed**; **2-residue ligand reassembled**	**Small “apparent ligand” removed**; **2-residue ligand reassembled**	PHQ removed and only DGL retained as the sole ligand
P24941	1GY3	A peptide ligand stored as a chain and a small-molecule ligand (ATP)	Correctly identify the chain as a ligand; identify ATP	**Chain ligand identified**; **ATP identified**	Chain ligand not identified; **ATP identified**	Resolution (2.7 Å) above the upper threshold	**Chain ligand identified**; **ATP identified**
Q13427	2WFJ	A peptide (cyclosporin A) stored as a chain	Identify one of the chains as the ligand, despite some of the residues being in the ATOM group and others in the HETATM group	**Chain ligand identified and fully rebuilt**	Chain ligand not identified	No valid ligands identified	**Chain ligand identified and fully rebuilt**
P00734	7KME	There are two distinct inhibitors bound to the protein	Identify the two ligands and find their corresponding PRD IDs to provide additional support	**Both ligands identified**	Only one ligand identified	**Both ligands identified**	**Both ligands identified**
P00533	1MOX	Glycosylated protein with no co-crystal ligands	Correctly identify the oligosaccharides listed in the PDB that are not ligands	**No ligands identified**	Oligosaccharides incorrectly identified as ligands	**No ligands identified**	**No ligands identified**
Q15910	5LS6	The structure contains a typical small-molecule ligand (74D) and an 11-residue chain (Jarid2 K116me3) that also appears to be a ligand	Correctly identify 74D and Jarid2 K116me3 (chains Q/R/S/T) as ligands	**74D and Jarid2 K116me3 correctly identified**	**74D correctly identified**; Jarid2 K116me3 incorrectly discarded	Resolution (3.47 Å) above the upper threshold	**74D and Jarid2 K116me3 correctly identified**
P00734	1AI8	A natural small protein in the complex (the alpha-thrombin light chain), bound to a peptide ligand and a small molecule (T42)	Do not identify chain L as a peptide ligand, despite its small size falling within the typical peptide range; identify chain I as a peptide ligand; identify T42 as a ligand	**Chain L correctly discarded as a peptide**; chain I not identified as a ligand; **T42 identified as a ligand**	**Chain L correctly discarded as a peptide**; chain I not identified as a ligand; **T42 identified as a ligand**	No ligands identified	Chain L incorrectly identified as a peptide; **chain I and T42 correctly identified as ligands**

*Note*: A subset of challenging PDB structures was selected for in-depth manual inspection to compare the performance of LigExtract *versus* ProteinsPlus, MOAD, and BioLiP2. Text in bold indicates a correct ligand idenfication decision. PDB, Protein Data Bank.

**Figure 3:**
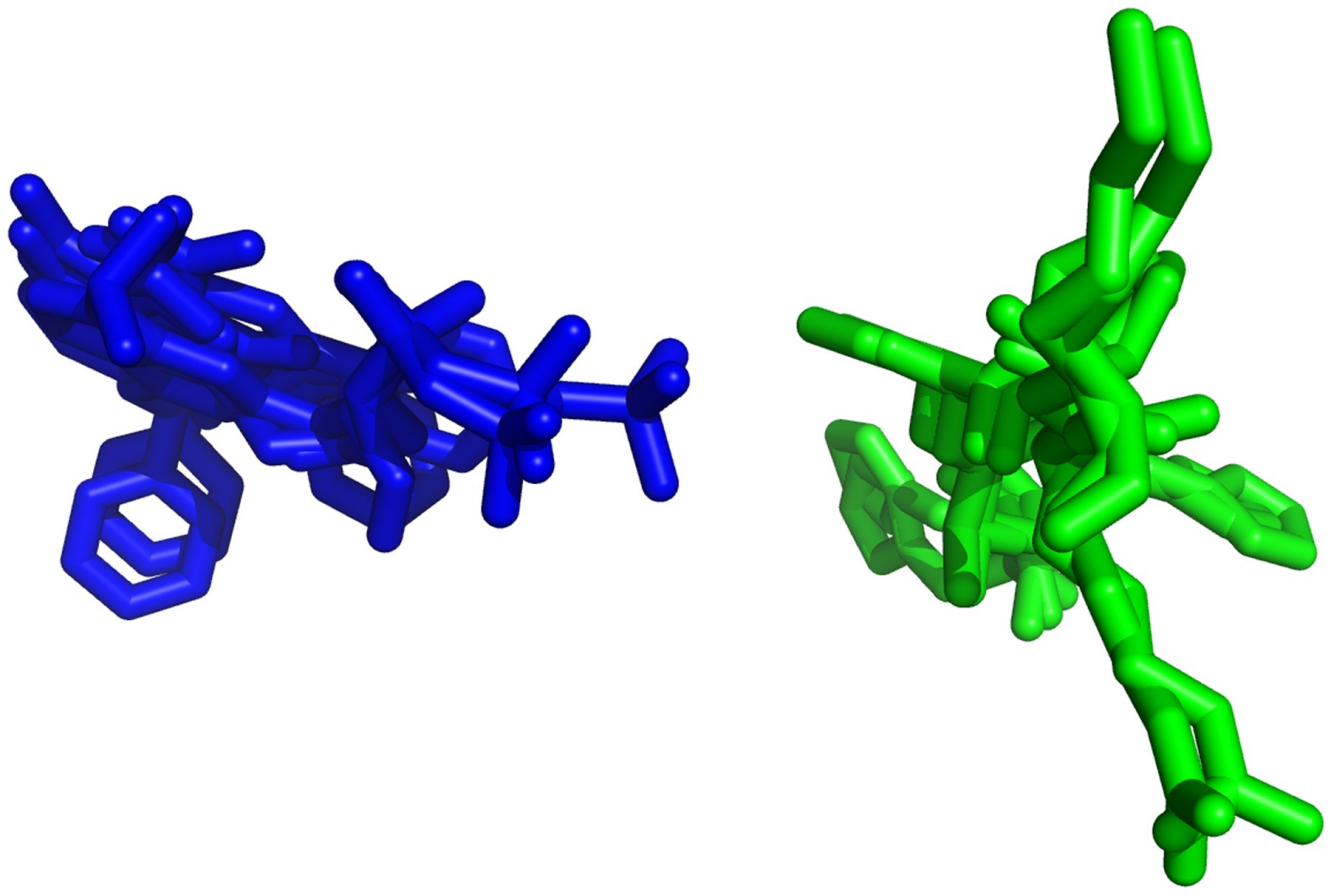
Image of ligand clusters generated by the *cluster* mode The *cluster* mode spontaneously identified two distinct ligand clusters (represented in blue and green, respectively) from a total of 16 ligands originating from 5 different PDB entries selected from the UniProt query P24941 (listed in the “challenging” benchmark dataset).

Regarding the *cluster* mode, instead of an attempt to deduplicate equivalent ligands, all eligible ligands are retained and organized in terms of (tentative) binding clusters (which may or may not be in separate binding sites). This is achieved by aligning PDB structures of each query protein using PyMOL (v3.0.0, open-source). The alignment quality is assessed by using the root mean square deviation (RMSD) values output by PyMOL, and PDB structures with RMSD > 3.5 Å are excluded with a warning recorded in the log file. Alignment is performed against the first PDB file in the name-sorted list of files, and only chains in the vicinity of at least one ligand are retained for the alignment (ligands selected by the identification procedure are retained inside their chains). After the alignment is completed, all ligand centroids are obtained by calculating the mean values for X, Y and Z coordinates for each ligand. Finally, the centroids for each protein query are grouped using hierarchical clustering with the Agglomerative Clustering function in scikit-learn (sklearn.cluster.AgglomerativeClustering). This function uses Euclidean distance (where 1 unit of distance corresponds to 1 Å) and a distance threshold of 10 Å to prevent merging pairs of clusters separated by a distance greater than 10 Å. A file is then generated with a breakdown of the obtained clusters, their ligand content, and their centroids, accompanied by figures where all clusters are shown together and the molecules are color-coded according to cluster membership (example in **Figure 3**). This allows users to quickly check whether some clusters should be merged or separated, or if incorrect assignment has been made.

At the end of both *cluster* and *filter* modes, after all processing, a tab-separated file is created containing all identified ligands (their file and ligand type) in each PDB file. This file is additionally annotated with the corresponding resolution, UniProt ID, chains matching the protein of interest, all chains contacting with the ligand, and the number of residues in the vicinity of the ligand.

Depending on the purpose for which the end user needs the list of ligands for the proteins of interest, either the *filter* or the *cluster* mode will be more helpful. For large-scale studies where the user is only interested in, for example, studying all ligands co-crystalized with a set of proteins, the *filter* mode can be employed. Alternatively, if the user is interested in carrying out large-scale docking studies or if the goal is to enumerate all different binding sites covered by X-ray structures, the *cluster* mode should be used.

### Performance comparison of LigExtract and existing tools for challenging ligand extraction

To highlight the challenges of automated ligand extraction, we selected seven representative examples that were identified during the development of LigExtract, which were later incorporated into the “challenging” benchmark dataset. Table 2 shows the ligand extraction results of LigExtract, ProteinsPlus, MOAD, and BioLiP2.

**Figure 4:**
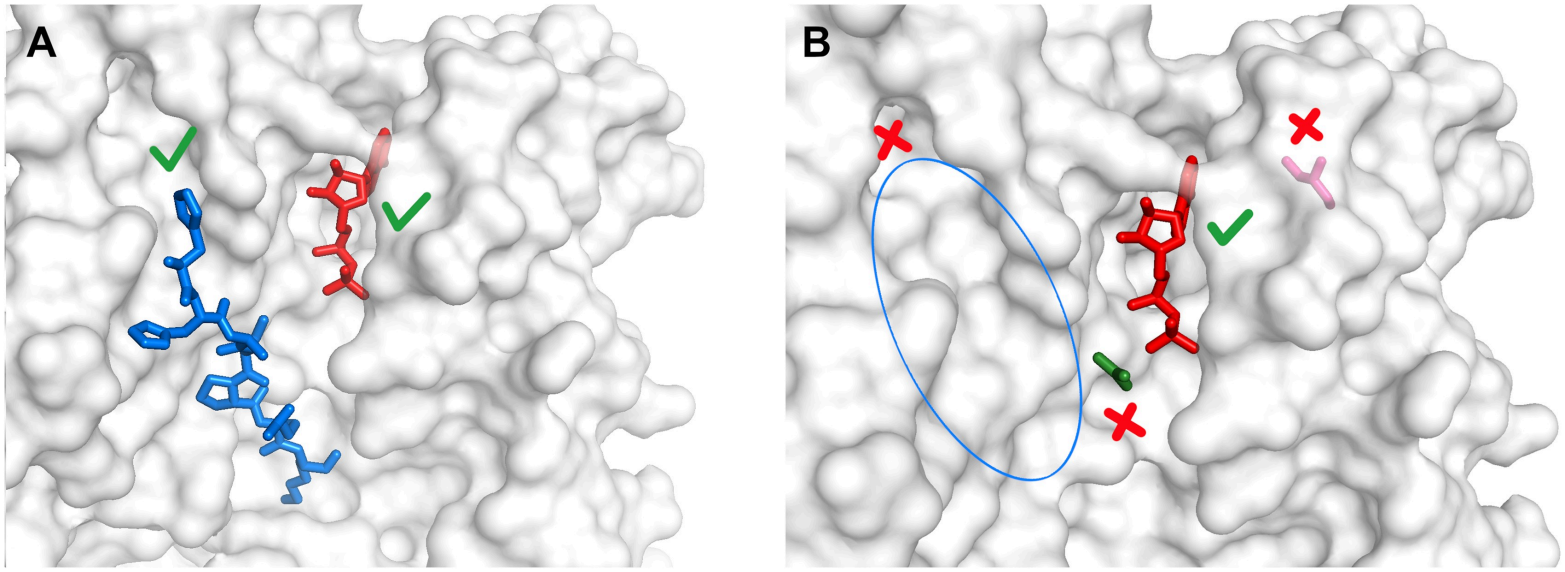
Ligand identification in PDB 1GY3 **A**. Correct ligand identification performed by LigExtract. **B**. Incorrect ligand identification performed by ProteinsPlus. The two ligands in PDB 1GY3 are a peptide ligand (blue) and a small-molecule ATP (red). The green check mark, red cross mark, and blue ellipse represent correct identification, incorrect identification, and failed ligand identification, respectively.

PDB 1KR6 contains an apparent ligand with the code PHQ, which is actually a fragment of a larger peptide ligand. In this case, LigExtract, ProteinsPlus, and MOAD correctly identified the full-length peptide ligand and excluded PHQ. However, BioLiP2 identified no ligands. PDB 7KME has two peptide inhibitors (in chains I and J), which were correctly identified by LigExtract, BioLiP2, and MOAD. However, ProteinsPlus only detected one of them (chain J). PDB 1MOX has multiple apparent ligands, which are actually *N*-glycosylation groups in the protein, so this PDB structure actually has no ligands. LigExtract, BioLiP2, and MOAD correctly identified no ligands, whereas ProteinsPlus identified multiple (five) false-positive ligands. PDB 1GY3 contains two ligands: a peptide ligand (chain E/F) and a small molecule (ATP). Both were identified by LigExtract (**Figure 4**A) and BioLiP2. ProteinsPlus identified ATP but also identified three other non-ligands, and failed to identify the peptide ligand (Figure 4B). MOAD excluded this PDB due to its resolution > 2.5 Å.

**Figure 5:**
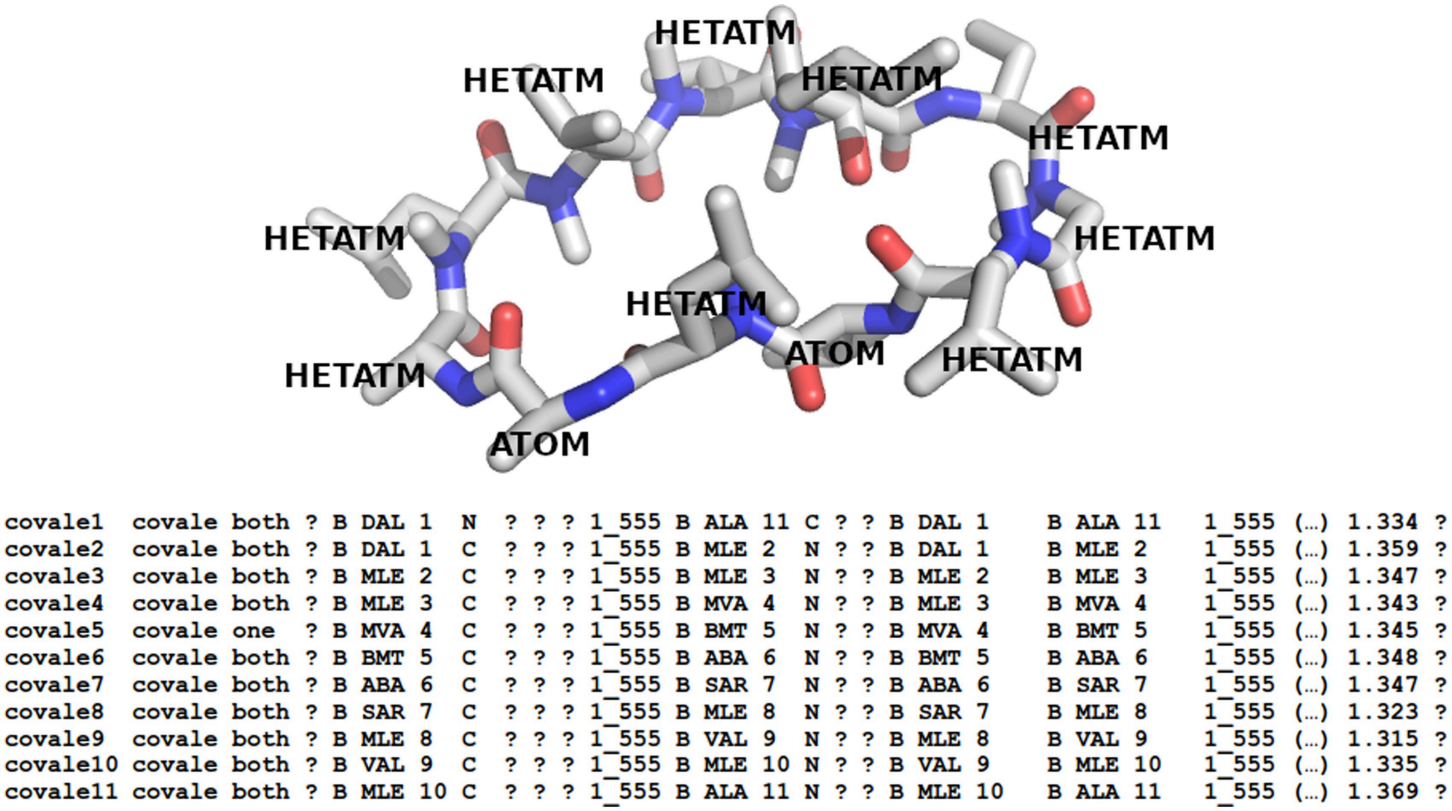
Cyclosporin A in PDB 2WFJ Cyclosporin A (chain B) consists of residues annotated as ATOM intercalated with residues annotated as HETATM. The full structure can only be reliably rebuilt from the information in the *_struct_conn* section of the PDB.

PDB 2WFJ features a protein complexed with cyclosporin A, which is represented as a chain and was only identified by LigExtract and BioLiP2. This ligand presents an additional challenge as it is composed of 11 residues, some of which are recorded in the HETATM group and others in the ATOM group within the *_atom_site* category. While residues with sequential residue numbers in the ATOM group are assumed to be connected, the same does not apply to those in the HETATM group (**Figure 5**). Therefore, we implemented a routine that exhaustively searches the connections annotated in the *_struct_conn* category to reassemble the full ligand structure such as in the case of cyclosporin A in PDB 2WFJ (Figure 5). This routine uses the NetworkX Python package [32] to construct a connectivity graph from all connections present in the *_struct_conn* category. As this category provides information for both covalent and non-covalent bonds, we only consider the bonds with the “covale” annotation.

**Figure 6:**
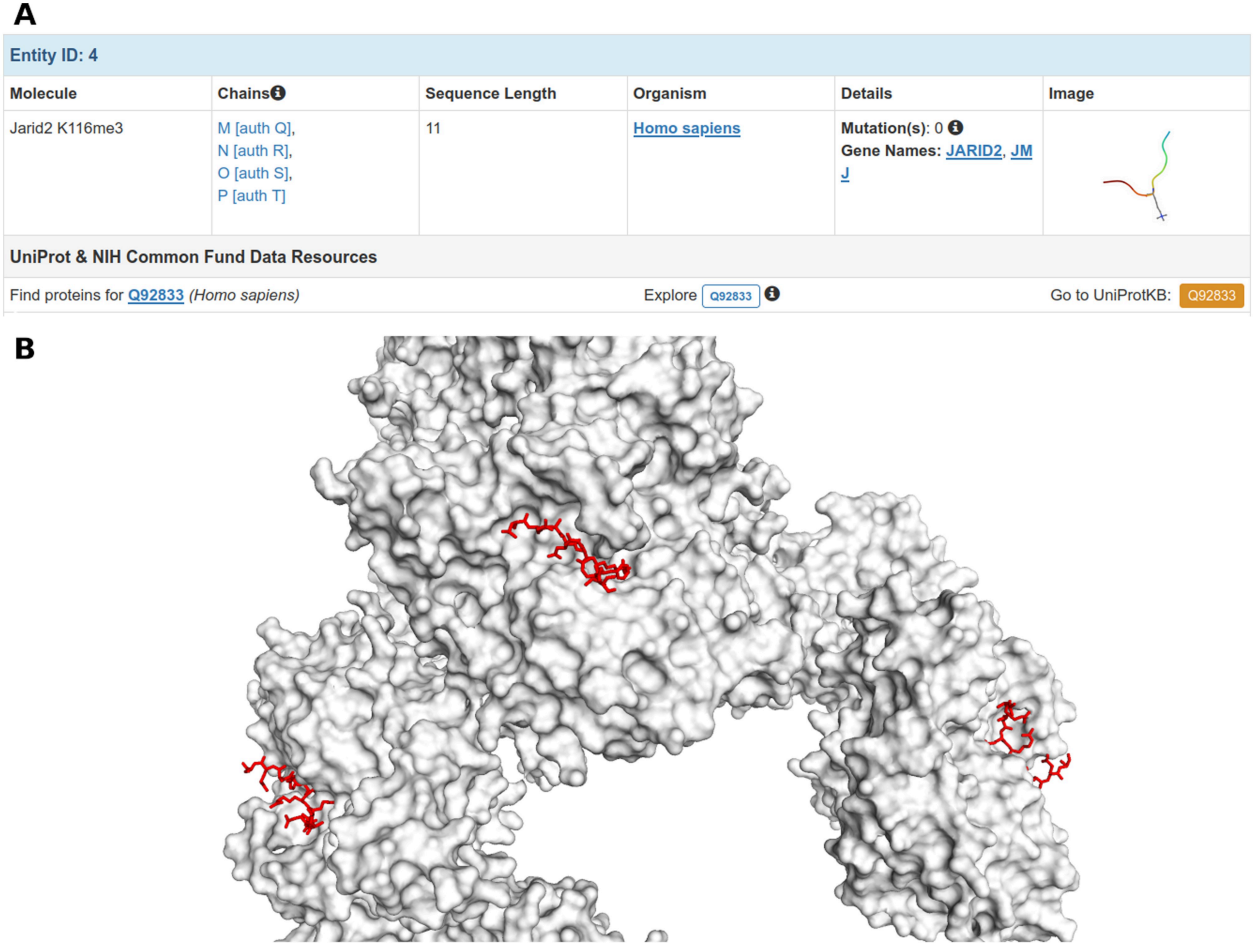
Jarid2 K116me3 is likely a ligand in PDB 5LS6 **A**. Annotation of Jarid2 K116me3 as four chains on the PDB website. **B**. Jarid2 K116me3 bound to different sites on the protein.

In PDB 5LS6, there are two ligands: a small molecule (74D) and a peptide ligand (Jarid2 K116me3), the latter of which is identified as four separated, repeated chains (**Figure 6**A). Although this peptide ligand is not explicitly annotated as a ligand in this particular structure deposition, and the original publication refers only to an “H3K27me3 activator peptide”, other work [33] describes “Jarid2-K116me2/3” as a peptide that binds to EED (a protein in 5LS6) to activate this protein. Furthermore, Jarid2 K116me3 is spread throughout a larger protein chain, a pattern typically consistent with a ligand rather than a protein (Figure 6B). Therefore, Jarid2 K116me3 is most likely a ligand which could be regarded in a similar light as ATP: not the main focus of the study in which it was reported, but still a legitimate ligand. And much like ATP, depending on a particular researcher’s goal, considering Jarid2 K116me3 could be of interest. Both LigExtract and BioLiP2 identified the small-molecule ligand as well as the peptide ligand, while MOAD excluded this PDB entry during curation due to its resolution exceeding the upper threshold enforced by MOAD curators.

The last case in Table 2, PDB 1AI8, illustrates well the difficulty in accurately detecting peptide ligands. In this structure, a peptide called “hirudin IIIB” (chain I) is a peptide ligand, while “alpha-thrombin (small subunit)” (chain L) is a protein. However, both fall within the same size range (under 40 residues, a range consistent with that of peptides), both are mapped to a UniProt ID, and both are annotated as synthetic constructs. Additionally, both are annotated as *_entity.type* = “polymer” and *_entity.src_method* = “nat”, which means that they are classified as naturally occurring polymers. As previously discussed in the Introduction section, enforcing a size-based cutoff is not a good solution. Therefore, besides reading the original publication, there is no obvious set of rules to distinguish between the two. Consequently, we could either identify both as peptides or neither. In this case, LigExtract and ProteinsPlus identified neither as a ligand, while BioLiP2, which uses a simple size cutoff, identified both as ligands. Additionally, LigExtract, ProteinsPlus, and BioLiP2 also identified the small molecule T42 in PDB 1AI8 as a ligand, while MOAD identified no ligands.

### Performance benchmarking of LigExtract against existing ligand extraction tools

We first benchmarked the performance of LigExtract against three other tools (ProteinsPlus, MOAD, and BioLiP2) using the “easy” benchmark dataset (*n* = 25 PDB entries), by measuring precision (*i.e.*, the proportion of selected ligands that are legitimate ligands) and recall (*i.e.*, the proportion of legitimate ligands that are successfully retrieved as ligands). As shown in **Table 3**, LigExtract achieved both perfect precision (100%) and recall (100%), which was expected given that the “easy” benchmark dataset was composed only of straightforward PDB entries, typically with only one protein and only small-molecule ligands stored in the HETATM group (*i.e.*, where a typical ligand would be found). Also, this dataset was assembled during the development of LigExtract. MOAD showed lower recall (81%) as it failed to identify five true ligands, such as colchicine (code LOC) in PDB 6AJZ. MOAD also showed lower precision (95%) as it incorrectly identified BEN (previously named BAM) as a ligand in PDB 2ANY. This compound is not a true ligand of interest as it is benzamidine, a known crystallization additive. ProteinsPlus also showed poorer precision than LigExtract (38% *versus* 100%) despite achieving perfect recall (100%), indicating that ProteinsPlus successfully identified all true ligands across 25 PDB entries but also identified additional non-ligands. It is important to emphasize that, unlike MOAD, ProteinsPlus (Protoss) is not designed specifically for ligand extraction; its main function is to predict the protonation states of protein–ligand complexes. BioLiP2 also showed lower precision than LigExtract (82% *versus* 100%) as some undesired additional ligands were identified, but it successfully retrieved all true ligands of interest (recall = 100%).

**Table 3:** Ligand extraction performance on the “easy” benchmark dataset

Tool	**Mean precision** **(± SD)**	Mean recall (± SD)
LigExtract	**100%** (± 0)	**100%** (± 0)
ProteinsPlus	38% (± 40%)	**100% **(± 0)
MOAD	95% (± 21%)	81% (± 38%)
BioLiP2	82% (± 34%)	**100%** (± 0)

*Note*: The mean precision and mean recall refer to the mean precision and recall values across all PDB entries (*n* = 25) in the “easy” benchmark dataset. The best performance is highlighted in bold. SD, standard deviation.

We then benchmarked the performance of LigExtract against ProteinsPlus, MOAD, and BioLiP2 using the “challenging” benchmark dataset (*n* = 107 PDB entries). As shown in **Table 4**, LigExtract achieved the highest mean recall (92%), performing significantly better than ProteinsPlus (64%) and MOAD (36%) and slightly better than BioLiP2 (91%). This result indicated that LigExtract was the most effective tool at retrieving “true” ligands of interest among the identified ligands. The sources of error in LigExtract will be discussed in detail in the next section. A recall of 64% from ProteinsPlus indicated that in approximately one-third of the PDBs, at least one legitimate ligand was missed. MOAD showed very low recall (36%), which can be largely attributed to the exclusion of 60 PDB entries due to the absence of detected ligands, resolution above 2.5 Å, or the presence of more than 4 nucleotides. One PDB query was also excluded from MOAD after “visual inspection” (as described on the MOAD website; no further details were provided). When evaluating precision on the “challenging” benchmark dataset, ProteinsPlus, MOAD, and BioLiP2 all showed lower performance (52%, 86%, and 75%, respectively) than LigExtract (100%). These results indicated that the three competing tools identified a variety of additional non-ligands alongside the true ligands. The additional molecules are, for example, crystallization additives, solvent molecules, or ions. We would like to reiterate that identifying additional ligands such as ions is not formally incorrect, it is simply not useful when one is looking to retrieve just organic compounds of interest (*i.e.*, drug-like molecules). Thus, a tool like BioLiP2 remains valuable when researchers aim to include all ligands with a biological role.

**Table 4:** Ligand extraction performance on the “challenging” benchmark dataset

Tool	Mean precision (± SD)	Mean recall (± SD)
LigExtract	**100%** (± 0)	**92%** (± 20%)
ProteinsPlus	52% (± 40%)	64% (± 38%)
MOAD	86% (± 34%)	36% (± 40%)
BioLiP2	75% (± 31%)	91% (± 27%)

*Note*: The mean precision and mean recall refer to the mean precision and recall values across all PDB entries (*n* = 107) in the “challenging” benchmark dataset. The best performance is highlighted in bold.

We purposefully included one of the most challenging scenarios in ligand detection — the presence of a fragment derived from a known protein, hirudin (examples are provided below and in Table S1). The challenge in this case stems from the fact that such a fragment is a short peptide sequence annotated in a way that makes it, in many instances, indistinguishable from a naturally occurring small protein, as discussed earlier. In the case of hirudin, when the peptide was used as an inhibitor, authors annotated the chain with names like “hirugen” or “hirulog”, making it fairly easy to recognize by name. However, one can quickly realize that a dictionary of such peptide names would have to be assembled to extend this type of ligand identification to all proteins (a dictionary that would need constant manual updates). Furthermore, these names are not always used; instead, the name of the protein from which the peptide derives is used. For this very reason, LigExtract was able to identify some instances of hirudin-derived peptides but not the majority.

In the case of PDB 1C5O, for example, the original research paper [34] clearly states that acetyl-hirudin (a known peptide inhibitor) was co-crystallized with thrombin, with and without additional small-molecule inhibitors. However, two aspects of the acetyl-hirudin annotation might misleadingly suggest that it is the full-length protein (hirudin) from which this peptide inhibitor derives: (1) it is named hirudin, and (2) it is associated with a UniProt ID. It should be noted that the original documentation and structure deposition instructions from RCSB (http://www.wwpdb.org/documentation/procedure; accessed 14 April 2022) indicate that UniProt ID mapping is automatically offered based on the sequence provided, and authors should typically select among the mapped IDs offered. Furthermore, this molecule is annotated as a natural polymer in the *_entity.type* and* _entity.src_method* categories, but it has no additional information in the *_pdbx_entry_details* category in the mmCIF file, which is reserved for depositors to clarify the nature of compounds in an entry (see examples in https://mmcif.wwpdb.org/dictionaries/mmcif_pdbx_v50.dic/Categories/pdbx_entry_details.html). This is understandable given that PDB 1C5O was deposited in 1999, long before the PDBx/mmCIF became the standard archiving format in 2014 (https://mmcif.wwpdb.org/docs/faqs/pdbx-mmcif-faq-general.html; accessed 14 April 2022). In contrast, the authors of PDB 1D6W, for example, used the same peptide (hirugen) but annotated the corresponding chain as “decapeptide inhibitor” and classified it as a synthetic polymer (in the *_entity* category). These two examples are a small illustration of how complex it can be to detect (even manually) whether or not a chain is a peptide ligand, which means that it is also complex to implement an automated detection procedure that covers all scenarios.

Unfortunately, as explained earlier, the presence or absence of UniProt ID mapping for a given chain in *_struct_ref_seq* cannot be used as a criterion to determine whether a chain is a peptide ligand or the corresponding protein, since there are instances where legitimate proteins lack any mapping to an external identifier, as well as instances where peptide ligands are mapped to a UniProt ID. To adopt a more conservative approach to build LigExtract, we consider all chains mapped to a UniProt ID as proteins by default, and implement a counter-check to determine whether certain chains have strong evidence of being a peptide ligand (see details in Module 2: Ligand Enumeration in the Method section). Beyond excluding chains with UniProt ID mappings from the ligand list, there is no way (other than manual inspection) to consistently and reliably distinguish complexed peptides from protein–protein interaction (PPI) complexes. In fact, the inherent nature of these elements in the PDB challenges the concept of “ligand”, as they can be used to simply mimic a PPI. In some cases, hirugen was identified by LigExtract because the depositing authors either annotated it as a proper ligand (PDB 1ABJ) or represented it as a chain without any UniProt ID.

In the case of oligosaccharide ligands, their identification as legitimate ligands is also not straightforward. ProteinsPlus appears to consider an oligosaccharide a potential ligand if it is associated with a PRD ID, unlike BioLiP2. For example, in PDB entries 4KXA and 4KX8, two oligosaccharide chains (chains D and E) are associated with a BIRD ID but are covalently bound to the protein. The original publication [35] makes no mention of these oligosaccharides, suggesting that these are simply glycosylation groups unrelated to the study. Nevertheless, due to their presence in the BIRD database, we prefer to classify such groups as potential ligands, accompanied by a warning in the extraction log file to inform users that this particular ligand is covalently bound to the protein. This allows for straightforward identification of such ligands, therefore avoiding the unwanted pooling of covalent and non-covalent ligands in specific analyses.

Although the “challenging” benchmark dataset was intentionally assembled to include difficult cases of ligand identification, including cases that are not yet properly addressed by our tool, both this and the “easy” benchmark dataset were assembled before LigExtract was in its current state and guided its development. Therefore, we benchmarked the performance of LigExtract against ProteinsPlus, MOAD, and BioLiP2 using the “random” benchmark dataset, an unbiased dataset consisting of 20 proteins from a pool of 673 well-known proteins described by Oprea and colleagues [24]. LigExtract showed near-perfect ability to identify all ligands, outperforming existing tools in terms of overall balance between precision and recall (98% *versus* 91.5% for the closest competing tool; **Table 5**). The only imprecision by LigExtract was the incorrect identification of a small-molecule ligand (5HD in 3DWB) and a peptide ligand (chain B in 5G1X). Upon inspecting the corresponding research article, 5HD is likely not a ligand of pharmaceutical interest, but even the original publication is not clear in this regard. This is likely a crystallization additive, but all information regarding 5HD makes it appear like a ligand.

**Table 5:** Ligand extraction performance on the “random” benchmark dataset

Tool	Mean precision (± SD)	Mean recall (± SD)	Balanced accuracy
LigExtract	98% (± 11%)	98% (± 11%)	98%
ProteinsPlus	61% (± 36%)	94% (± 23%)	77.5%
MOAD	**100% (± 0)**	78% (± 40%)	89%
BioLiP2	83% (± 29%)	**100% (± 0)**	91.5%

*Note*: The mean precision and mean recall refer to the mean precision and recall values across all PDB entries in the “random” benchmark dataset (*n* = 20). Balanced accuracy is the average between precision and recall. The best performance is highlighted in bold.

MOAD, being manually curated, showed 100% precision but much lower recall (failing to identify some true ligands). In contrast, BioLiP2 showed 100% recall but much lower precision (identifying ligands that are not true ligands of interest, such as Zn^2+^, Mg^2+^, and 5HD in 3DWB).

### Cases of compromised performance in LigExtract

There were a total of 16 cases of PDB structures with undetected ligands, 15 corresponding to the same protein query (P00734). In all the 15 cases, there was a single undetected ligand per PDB, corresponding to a hirudin peptide derivative encoded as a protein chain (taking various names such as “hirulog 3” or even the name of the protein from which it derives — hirudin). As discussed earlier, automated identification of these “chain ligands” is practically impossible and would require manual inspection of the underlying publications. To aggravate the complexity entailed in distinguishing between a protein fragment and a legitimate peptide ligand, in PDB 1C5L, the ligand is even named hirudin — the same name as the full-length protein from which this peptide derives. Nevertheless, when PDB annotations are not ambiguous, as in this exception, peptide ligands are identified by LigExtract, which we will discuss next. The remaining case corresponds to PDB 5G1X, already described.

### Correctly identifying peptide ligands

As previously mentioned, in a PDB structure, peptide ligands can belong to the HETATM group, the ATOM group, or both. At first glance, segments of other proteins complexed with the query protein might be incorrectly perceived as peptide ligands or might indeed function as legitimate ligands. For example, in PDB 7KME, the protein alpha-thrombin is complexed with two molecules, hirugen (chain I) and SEL2711 (chain J). While the focus of this particular crystal structure is to study the novel SEL2711 inhibitor, hirugen is also an inhibitor of alpha-thrombin. MOAD, which relies on manual curation, identifies these two molecules as ligands, but ProteinsPlus identifies only SEL2711. In LigExtract, we combine these two outcomes by notifying users that both ligands exist but SEL2711 is additionally a “biologically interesting molecule” (*i.e.*, present in BIRD). This approach informs users of the two possible binding sites and allows making an informed decision on how to proceed.

LigExtract differentiates peptide ligands (desired) from segments of other proteins (undesired) that may be complexed with the protein of interest by inspecting where proteins are annotated with a UniProt ID and “non-protein” polymers are not (*e.g.*, PDB 6U4Y). However, there are instances (*e.g.*, PDB 5HGV) where a segment of a protein exists in the X-ray structure but is not annotated with the corresponding UniProt ID. To differentiate these instances from cases where a peptide is a true ligand, LigExtract uses the PDBe REST API, where additional annotation is available.

### Correctly identifying PDB entries where the provided UniProt protein does not participate in the binding site

If a user is interested in studying all PDB entries for a given protein, it is important to ensure that this protein plays a key role in each PDB retrieved. There are straightforward cases such as CSNK2A1 (UniProt ID: P68400), where a UniProt search using the accession number easily reveals that, for example, PDB 5HGV has only 13 residues for protein P68400. However, multiple other less clear cases exist where the protein of interest occupies a large portion of the PDB entry but does not participate in the binding site. This can be observed when searching for EZH2: the query returns a number of PDB entries that have a large number of EZH2 residues, but in which the ligand is actually bound to another protein. LigExtract is capable of flagging these cases to avoid incorrect grouping of PDB structures of a protein that is bound to an inhibitor, and this is achieved by detecting whether the protein of interest has any residues within 6 Å of any ligands.

### Addressing the issue of “incomplete” or disconnected ligands

If a ligand is composed of amino acid groups bound to non-amino acid groups, the annotation of the former in the ATOM group and the latter in the HETATM group may create the illusion that a given non-amino acid group is an entire ligand, especially when using the PDB-to-ligand mapping within PDB. One instance of this phenomenon is the PHQ ligand which is listed as one of the ligands in up to 12 structures, although PHQ is in fact only a part of a larger ligand, as discussed earlier. Other examples of such “incomplete” ligands are MCM (*e.g.*, PDB 3EWF) and SLL (*e.g.*, PDB 5XHS).

LigExtract addresses this issue by checking whether an identified ligand is bound to additional atoms in a non-protein chain.

### Additional LigExtract features

Some other potentially useful features of LigExtract are: (1) Flagging cases in which one or more ligands are present but none interact with the protein query; (2) Resolving atoms with alternate occupancy; (3) Cleaning the PDB structure to retain only proteins that interact with any ligand; (4) Considering chimera proteins as valid instances of the UniProt ID query as long as part of the chimera is the protein query (even if this part does not contact the ligand).

LigExtract relies on multiple open-source tools for different tasks, beyond general Python libraries such as numpy, pandas, scipy, and scikit-learn, namely: PyMOL (v2.0; https://pymol.org)⁠, BioPandas [36], NetworkX [32], and RDKit (open-source cheminformatics; http://www.rdkit.org).

## Conclusion

The PDB is a key resource for drug discovery, currently storing hundreds of thousands of structures. At this scale, manual curation is infeasible; therefore, automated approaches are required to enable researchers to draw insights from existing structures (whether liganded or not). This is particularly important for the identification of ligands of pharmaceutical interest. There are a few tools that allow automated processing of structures in PDB to extract ligands; however, due to the high nuance of the problem, these tools do not handle a range of ligand annotation scenarios. To address this issue, we developed LigExtract, a tool specifically designed for the identification, curation, and extraction of ligands.

To build this tool, we manually explored a large number of PDB entries to understand how depositors typically annotate a PDB entry. In this process, we specifically focused on cases where there are non-conventional ligands, such as peptides and oligosaccharides), for which identification has no clear rules. Additionally, we manually curated a list of crystallization additives and other molecules/atoms of that nature to facilitate the identification of true ligands of biological interest. To benchmark how LigExtract performs in comparison to other existing tools, we constructed three datasets: the “easy” dataset with straightforward ligand identification, the “challenging” dataset where ligand identification is not obvious, and the “random” dataset constructed by a random selection of proteins after the tool was finalized.

Even though we purposefully included cases where automated ligand extraction would not be possible (*i.e.*, where identifying the ligand would require inspecting the literature accompanying the PDB entry), LigExtract still showed high precision and recall (100% and 92%) in the “challenging” dataset. Both metrics were significantly higher in LigExtract than in ProteinsPlus and MOAD, the two other tools where large-scale ligand identification is possible (at most 86% precision and 91% recall). BioLiP2 performed most similar to LigExtract, but it still is not suited to specifically identify organic compounds and “drug-like” molecules (which is the main purpose of LigExtract), as it identifies ligands such as ions.

LigExtract is the first tool specifically designed for large-scale automated identification of organic ligands of pharmaceutical interest and is, to date, the tool that addresses the most ligand annotation scenarios in the PDB.

## Code availability

All code is available on GitHub (https://github.com/comp-medchem/LigExtract), accompanied by instructions on how to install and run it.

## Supplementary Material

qzaf018_Supplementary_Data

## CRediT author statement

**Natália Aniceto:** Conceptualization, Investigation, Software, Methodology, Data curation, Formal analysis, Visualization, Writing – original draft, Writing – review & editing, Funding acquisition. **Nuno Martinho:** Methodology, Data curation, Validation, Writing – review & editing, Funding acquisition. **Ismael Rufino:** Data curation, Writing – review & editing. **Rita C. Guedes:** Conceptualization, Methodology, Supervision, Project administration, Funding acquisition, Resources, Writing – review & editing. All authors have read and approved the final manuscript.
