# Supplementary material for: LigExtract: Large-scale Automated Identification of Ligands from Protein Structures in the Protein Data Bank
Source: Genomics Proteomics Bioinformatics. 2025 Feb 28;23(4):qzaf018. doi: 10.1093/gpbjnl/qzaf018 (PMC12619641; doi:10.1093/gpbjnl/qzaf018)
Supplement: qzaf018_Supplementary_Data [file qzaf018_supplementary_data.zip › supplementary material captions.docx]

**Supplementary material**

**Table S1 The “challenging” dataset**

**Table S2 The “easy” dataset**

**Table S3 The “random” dataset**
